# Effect of the computer-aided static navigation technique on the accuracy of bicortical mini-implants placement site for maxillary skeletal expansion appliances: an in vitro study

**DOI:** 10.1186/s12903-023-02785-7

**Published:** 2023-02-11

**Authors:** Paulina Rodríguez Torres, Elena Riad Deglow, Álvaro Zubizarreta-Macho, Georgia Tzironi, Héctor González Menéndez, Juan Lorrio Castro, Ana Belén Lobo Galindo, Sofía Hernández Montero

**Affiliations:** 1 Department of Implant Surgery, Faculty of Health Sciences, Alfonso X El Sabio University, Avda. Universidad, 1, Villanueva de La Cañada, 28691 Madrid, Spain; 2 Department of Surgery, Faculty of Medicine and Dentistry, University of Salamanca, 37008 Salamanca, Spain; 3 Department of Dental Therapeutics, Faculty of Health Sciences, Alfonso X El Sabio University, 28691 Madrid, Spain

**Keywords:** Orthodontics, Mini-implants, Palatal expansion, Maxillary expansion, Maxillary skeletal expansion, Computer-aided navigation

## Abstract

The objective of the present study was to evaluate and compare the effect of the computer-aided static navigation technique on the accuracy of the maxillary skeletal expansion (MSE) appliances. Material and Methods: Forty orthodontic self-drilling mini-implants were placed in ten anatomically based standardized polyurethane models of a completely edentulous upper maxilla, manufactured using a 3D impression procedure. The four orthodontic self-drilling mini-implants for anchoring the MSE appliance were digitally planned on 3D planning software, based on preoperative cone-beam computed tomography (CBCT) scan and a 3D extraoral surface scan. Afterwards, the surgical templates were virtually planned and manufactured using stereolithography. Subsequently, the orthodontic self-drilling mini-implants were placed an postoperative CBCT scans were performed. Finally, coronal entry-point, apical end-point and angular deviations were calculated using a *t*-test for independent samples or a non-parametric Signed Rank test. Results: Statistically significant differences were not shown at coronal entry-point (*p* = 0.13), apical end-point (*p* = 0.41) and angular deviations (*p* = 0.27) between the planned and performed orthodontic self-drilling mini-implants. Conclusions: Computer-aided static navigation technique enables accurate orthodontic mini-implant placement for the MSE appliances.

## Background

Maxillary transverse deficiency is a common problem in orthodontics and may have several clinical manifestations such as posterior crossbite, dental crowding, protrusion of incisors, accentuated curve of Wilson, as well as dark triangles in the corner of the mouth [1]. This undesirable drawbacks have been commonly treated by rapid palatal expansion (RPE) procedures in growing patients [1–3]; RPE is a simple and predictable therapeutic approach with stable long and short term results. This therapeutic alternative has shown to be effective regardless of the type of maxillary expander used during primary, mixed and early permanent dentition [4]. However, adult patients require more invasive techniques such as surgically-assisted rapid maxillary expansion (SARME). In addition, Brunetto et al. [5] reported the efficacy microimplant- assisted rapid palatal expansion (MARPE) for expanding the maxilla of growing patients using orthodontic mini-implants. Maxillary Skeletal Expander (MSE) is comprised of bilateral bands attached to the upper molars and an expansion screw placed in palate with four welded tubes of 1.5 mm diameter and 2 mm length, that allow the insertion of the orthodontic self-drilling mini-implants, while the expander of MARPE device is constructed after the placement of the orthodontic self-drilling mini-implants; however, both procedures have reported to be effective in opening the mid-palatal suture in late adolescents and adult patients [6, 7]. Additionally, Hartono et al. recommended that the orthodontic self-drilling mini-implants should be 1.5 mm diameter and 11 mm length for a bicortical anchorage increasing stability of MSE appliances [8].

Additionally, the increasing popularity of orthodontic mini-implants has led to the development of different mini implant expansion techniques according to the preference of the clinician and the commercial availability [9]. However, the orthodontic mini-implants placement comes with a risk such as loss of stability, inflammation or infection, damage of anatomical structures, fracture of orthodontic mini-implants, failure of osseointegration etc. [10]. Therefore, the use of computer-aided navigation techniques has been suggested to improve the accuracy of orthodontic mini-implants placement [11, 12]. Specifically, the computer-aided navigation techniques consist in a therapeutic planning based on a cone beam computed tomography (CBCT) scan [13, 14] to evaluate the safest and most effective orthodontic mini-implant placement site in procedures of maxillary skeletal expansion (MSE) [15], since CBCT scan have shown to be much more accurate in assessing mini-implant placement site compared to orthopantomography scan or periapical radiographs [16].

The objective of the present study was to evaluate and compare the effect of the computer-aided static navigation technique on the accuracy of bicortical mini-implants placement site for MSE appliances, with a null hypothesis (H_0_) that there are no differences in the accuracy of the orthodontic self-drilling mini-implants placement for the MSE appliances.

## Methods

### Study design

Researchers conducted a controlled experimental trial between January to March 2022 at the Dental Centre of Innovation and Advanced Specialties at Alfonso X El Sabio University in Madrid, Spain. The Ethical Committee of the Faculty of Health Sciences at Alfonso X El Sabio University approved the study in December 2021 (process no. 2/2022). In addition, this study was conducted in accordance with the ethical guidelines established by the Declaration of Helsinki and the CONSORT Statement. The patient of 67 years old provided their informed consent for her preoperative CBCT scan to be used in this study. A power of 80.00% was calculated using the bilateral Student’s *t-*test for two independent samples. When used to calculate the variation from the null hypothesis H_0_: μ_1_ = μ_2_, the significance level of 5.00% and power of 80.00% meant that forty orthodontic mini screws were necessary for the purposes of this study.

### Experimental procedure

**Fig. 1:**
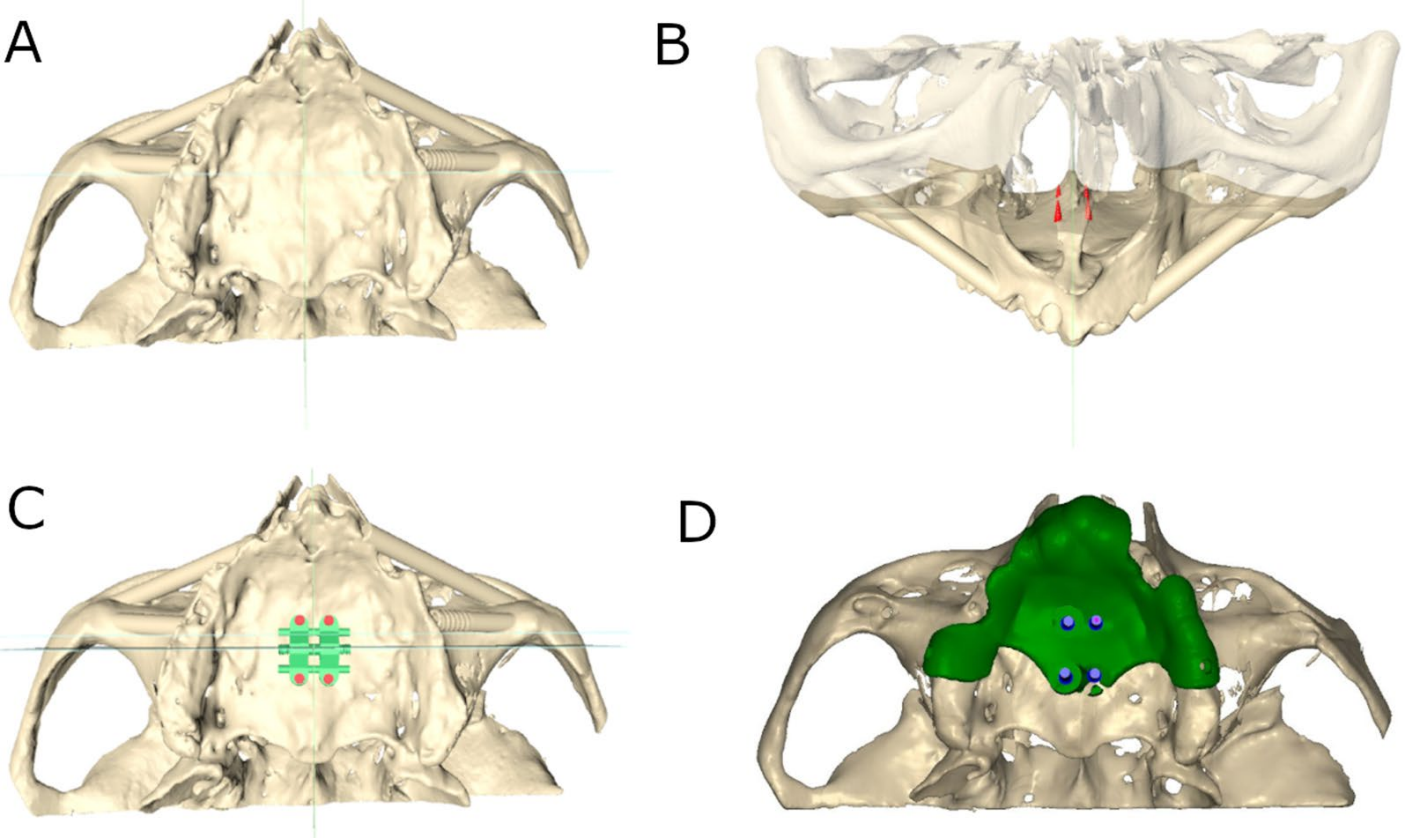
**A** Occlusal view of the preoperative digital file, **B** front view of the orthodontic self-drilling mini-implants virtually planned, **C** occlusal view of the SME appliance virtually planned and **D** surgical template virtually planned

Forty (40) orthodontic self-drilling mini-implants (Biomaterials Korea, Seoul, Republic of Korea) with 1.8 mm diameter and 9 mm length were planned and placed in ten (10) anatomically based standardized polyurethane models of a completely edentulous upper maxilla, manufactured using a 3D impression procedure (Objet30 OrthoDesk, Tikoa, Madrid, Spain) and based on a preoperative CBCT scan (WhiteFox, Satelec, Merignac, France). Moreover, the printing procedure consist of polymerizes a layer of support material (Ref.: SUP 705B, PolyJet Support Material, Stratasys, Canada) by means of head and lamp. The orientation of the STL digital files is automatically always leaving them as close as possible to the printing tray. There is no need to put it in any curing oven since this type of printer already photo-cures the guides during manufacturing. The CBCT scan was taken from a real patient using the following exposure parameters: 8.0 mA, 105.0 kV peak, 7.20 s, with a field of view of 15 mm × 13 mm. The use of polyurethane was based on the American Society for Testing and Materials’ (ASTM F-1839-08) approval of the use of polyurethane for testing instruments and dental implants ("Standard Specification for Rigid Polyurethane Foam for Use as a Standard Material for Test Orthopedic Devices for Instruments”) [17]. Subsequently, bicortical orthodontic self-drilling mini-implants and three fixation mini-implants (one anterior and two posterior) to the buccal cortical plate were virtually planned using 3D implant-planning software (Ortosan, Madrid, Spain) with the aforementioned measurements (Fig. 1A–C). Afterwards, the virtual templates were also designed (Fig. 1D) and manufactured using stereolithography (ProJet 6000, 3D Systems, Rock Hill, SC, USA).

**Fig. 2:**
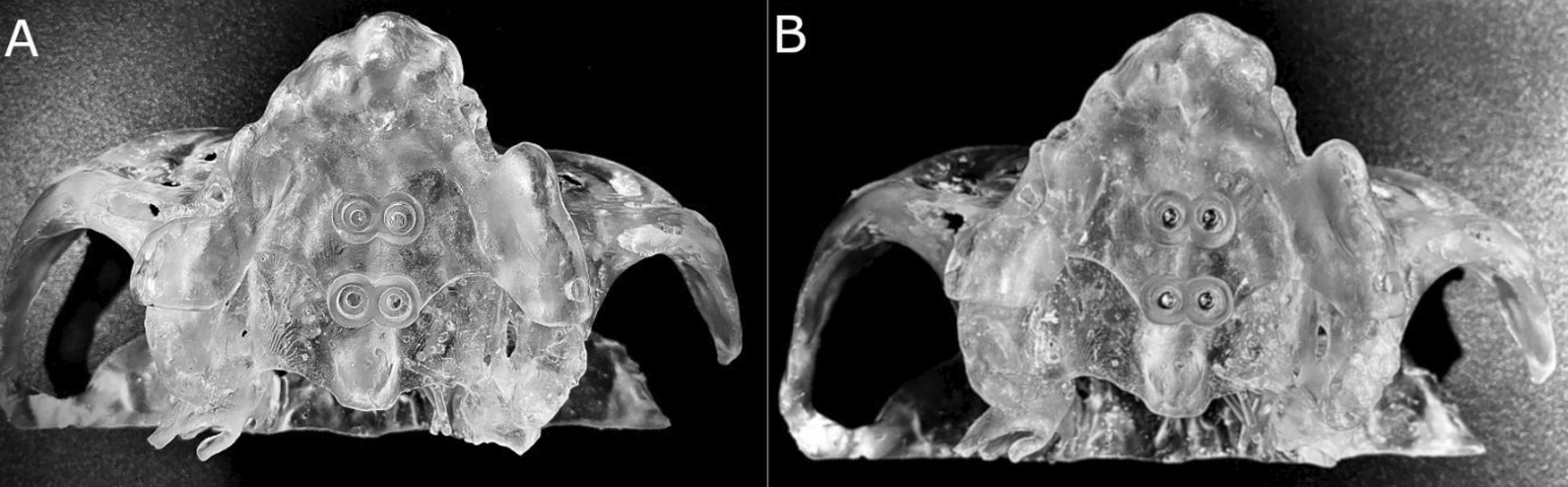
**A** Occlusal view of the anatomically based standardized polyurethane models of a completely edentulous upper maxilla with the stereolithographic surgical template and **B** orthodontic self-drilling mini-implants on the palate

The stability of all surgical templates was checked, and no adjustment was necessary (Fig. 2A).

Researchers randomized the orthodontic self-drilling mini-implants according to the placement site in palate (Epidat 4.1, Galicia, Spain), and assigned them to one of the following study groups: 1: orthodontic self-drilling mini-implants placed on the right posterior orifice of the surgical template (n = 10); 2: orthodontic self-drilling mini-implants placed on right anterior orifice of the surgical template (n = 10); 3: orthodontic self-drilling mini-implants placed on the left anterior orifice of the surgical template (n = 10); 4: orthodontic self-drilling mini-implants placed on the left posterior orifice of the surgical template (n = 10). The order of placement of the orthodontic self-drilling mini-implants was randomized across the study groups (Epidat 4.1, Galicia, Spain) (Fig. 2B).

All orthodontic self-drilling mini-implants were placed by a unique operator with prior surgical experience.

### Measurement procedure

**Fig. 3:**
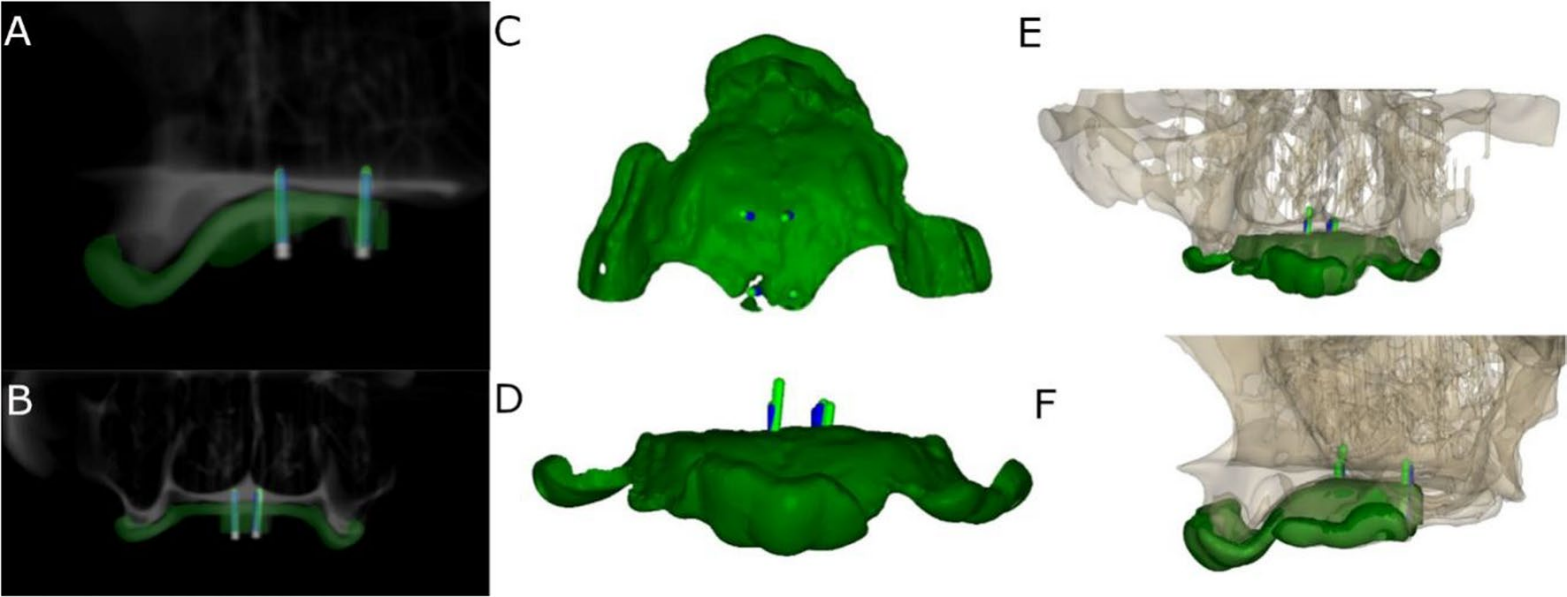
**A** Sagittal and **B** coronal view of the virtually planned (blue cylinders) and performed orthodontic self-drilling mini-implants (green cylinders) and surgical template (green line) on the CBCT scan, **C** top and **D** front view of the virtually planned (blue cylinders) and performed orthodontic self-drilling mini-implants (green cylinders) and surgical template and **E** front view and **F** sagittal view of the virtually planned (blue cylinders) and performed orthodontic self-drilling mini-implants (green cylinders) and surgical template in the completely edentulous upper maxilla

Following placement of the orthodontic self-drilling mini-implants, the researchers conducted postoperative CBCT scans (WhiteFox, Satelec, Merignac, France) using the aforementioned exposure parameters (Fig. 3A,B). The planning and postoperative CBCT scans (WhiteFox, Satelec, Merignac, France) of the different groups were subsequently imported into 3D implant-planning software (NemoScan, Nemotec, Madrid, Spain). Afterwards, the post-operative CBCT scan and the preoperative standard tessellation language (STL) digital file of the orthodontic self-drilling mini-implants planning were manually aligned by selecting the same anatomical key points of both the post-operative CBCT scan and the preoperative STL digital file by an unique operator using the 3D virtual implant-planning software (NemoScan, Nemotec, Madrid, Spain) so as to record the deviation, taken at the coronal entry point (mm), apical endpoint (mm), and angular deviation (°), the latter being measured in the center of the cylinder. This measurement procedure was used in a previous study to measure the deviations of conventional length dental implants [18]. If any deviations were noted in any of the orthodontic self-drilling mini-implants, an independent operator then analyzed and compared between axial, sagittal, and coronal views (Fig. 3C–F). Researchers also noted and analyzed the deviations in the position of orthodontic self-drilling mini-implants positions.

### Statistical tests

Statistical analysis was carried out using SAS 9.4 (SAS Institute Inc., Cary, NC, USA). The mean and standard deviation (SD) were used for descriptive analysis of quantitative data. For each of the variables, the difference between the pre- and post-values was analyzed using a *t*-test for independent samples or a non-parametric Signed Rank test based on compliance with the application criteria. *P* < 0.05 was determined as the level for statistical significance.

## Results

Table 1 shows the mean, median and standard deviation values for the coronal entry point (mm), apical end point (mm) and angular deviations (°) of each orthodontic self-drilling mini-implant.

**Table 1:** Descriptive values of deviations at the coronal entry point (mm), apical end point (mm), and angular (°) deviations of each orthodontic self-drilling mini-implant

Measure	IOI	*n*	Mean	SD	Median	Minimum	Maximum	*p*-value
Coronal	1	10	1.97	0.95	1.85	0.90	3.80	0.13
	2	10	2.72	0.77	2.70	1.70	4.00	
	3	10	2.60	1.40	2.30	0.20	5.20	
	4	10	1.92	0.72	1.95	0.80	3.50	
Apical	1	10	2.36	1.31	2.15	0.60	4.70	0.41
	2	10	2.65	0.85	2.65	1.60	4.00	
	3	10	2.86	1.26	2.30	1.30	5.40	
	4	10	2.16	0.70	2.10	1.30	3.80	
Angular	1	10	7.98	5.19	6.65	2.20	17.20	0.27
	2	10	7.46	6.71	4.75	0.90	19.50	
	3	10	5.64	1.64	5.70	2.70	8.30	
	4	10	4.83	1.75	4.60	2.30	8.60	

Group IOI 1: orthodontic self-drilling mini-implants placed on the right posterior orifice of the surgical template. Group IOI 2: orthodontic self-drilling mini-implants placed on right anterior orifice of the surgical template. Group IOI 3: orthodontic self-drilling mini-implants placed on the left anterior orifice of the surgical template. Group IOI 4: orthodontic self-drilling mini-implants placed on the left posterior orifice of the surgical template

**Fig. 4:**
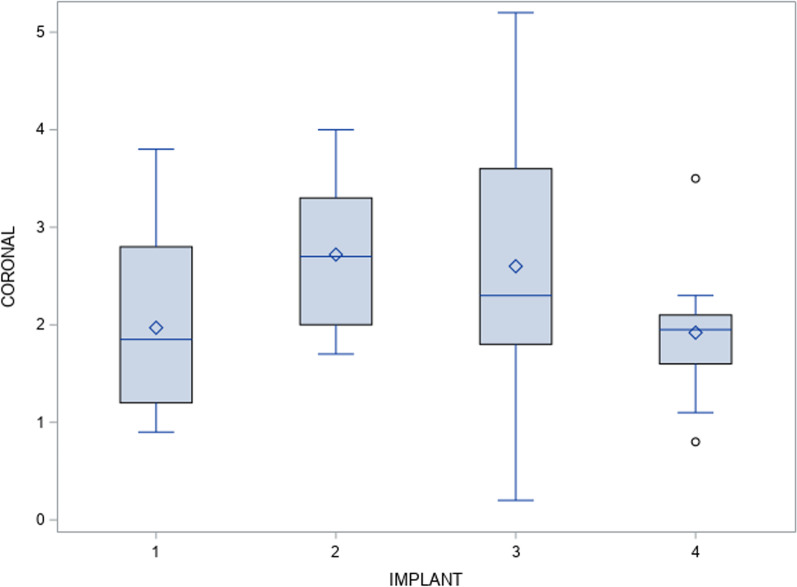
Box plot of the mean and SD values of each orthodontic self-drilling mini-implants at the coronal entry-point. The horizontal lines in each box represent the median values..◊ Mean value of the box plots. Coronal axis values in millimeters. Implant 1: orthodontic self-drilling mini-implants placed on the right posterior orifice of the surgical template. Implant 2: orthodontic self-drilling mini-implants placed on right anterior orifice of the surgical template. Implant 3: orthodontic self-drilling mini-implants placed on the left anterior orifice of the surgical template. Implant 4: orthodontic self-drilling mini-implants placed on the left posterior orifice of the surgical template

The paired *t*-test found no statistically significant deviations (*p* = 0.13) between the planned and actual surgical positions of the orthodontic self-drilling mini-implants placed through surgical template at the coronal entry-point (Fig. 4).

**Fig. 5:**
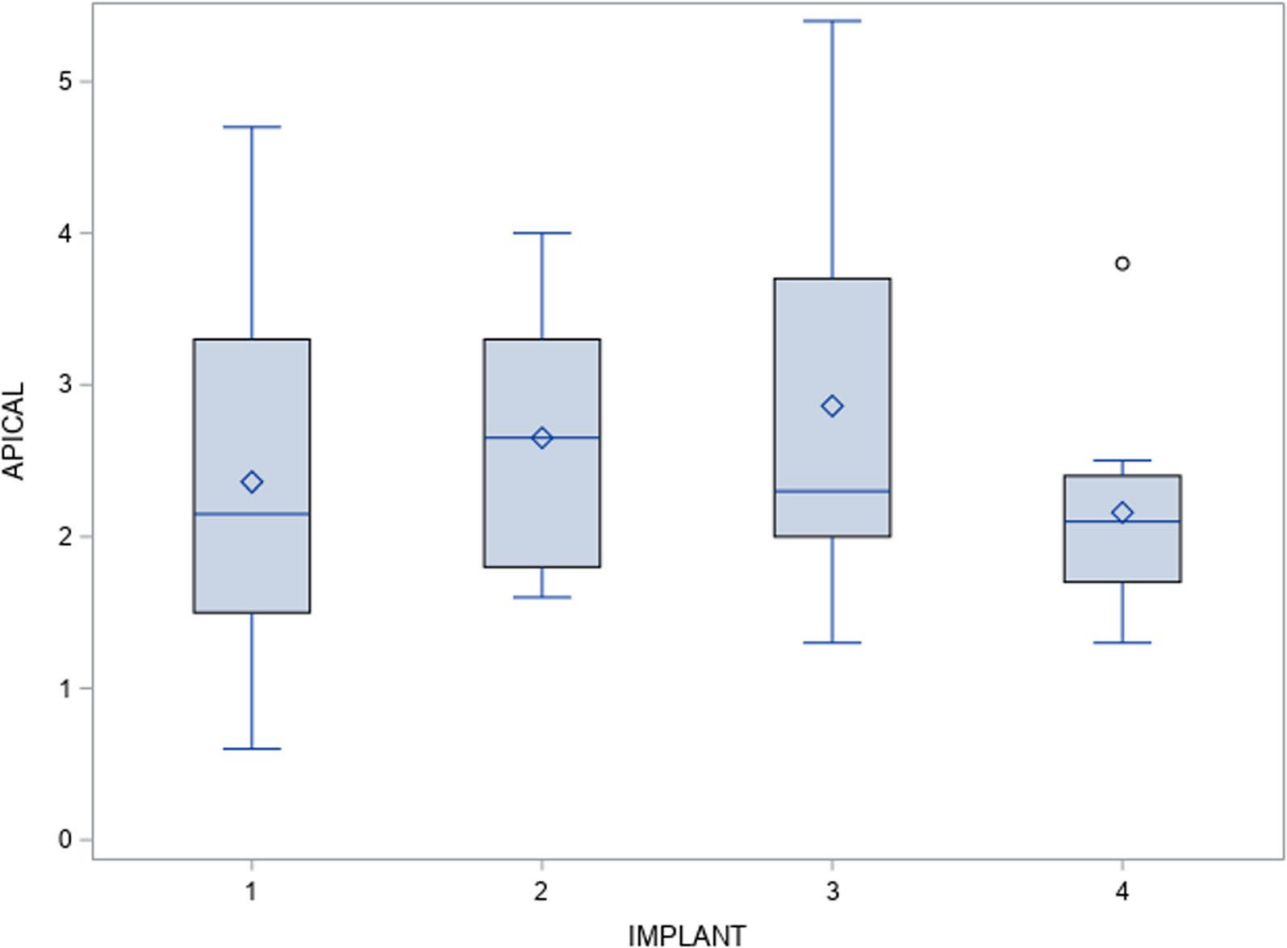
Box plot of the mean and SD values of each orthodontic self-drilling mini-implants at the apical end-point. The horizontal lines in each box represent the median values..◊ Mean value of the box plots. Coronal axis values in millimeters. Implant 1: orthodontic self-drilling mini-implants placed on the right posterior orifice of the surgical template. Implant 2: orthodontic self-drilling mini-implants placed on right anterior orifice of the surgical template. Implant 3: orthodontic self-drilling mini-implants placed on the left anterior orifice of the surgical template. Implant 4: orthodontic self-drilling mini-implants placed on the left posterior orifice of the surgical template

However, the paired *t*-test did not find any statistically significant deviations (*p* = 0.41) between the planned and actual surgical positions of the orthodontic self-drilling mini-implants placed through surgical template at the apical entry-point (Fig. 5).

**Fig. 6:**
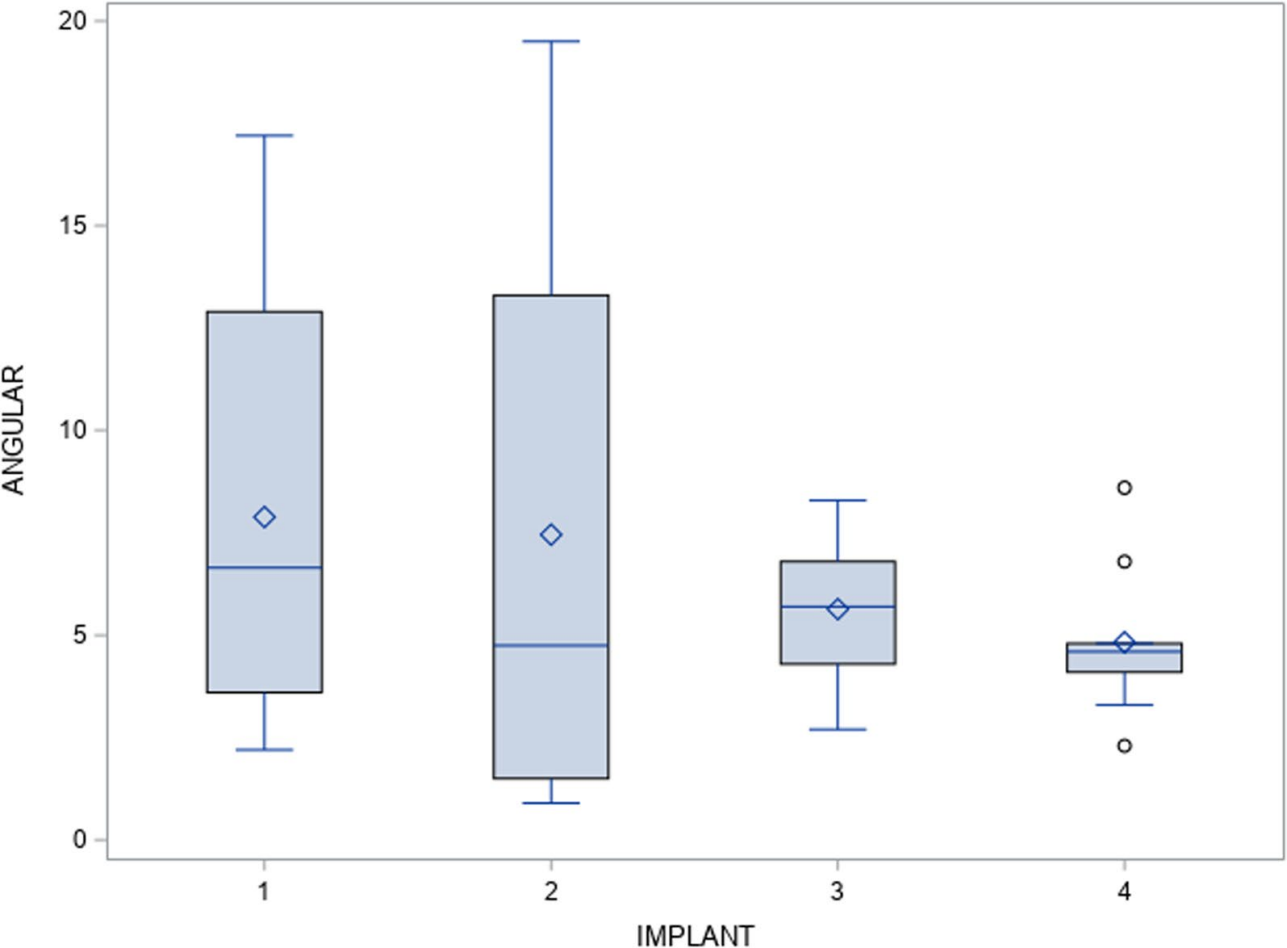
Box plot of the mean and SD values of each orthodontic self-drilling mini-implants at the angular deviations. The horizontal lines in each box represent the median values..◊ Mean value of the box plots. Coronal axis values in grades. Implant 1: orthodontic self-drilling mini-implants placed on the right posterior orifice of the surgical template. Implant 2: orthodontic self-drilling mini-implants placed on right anterior orifice of the surgical template. Implant 3: orthodontic self-drilling mini-implants placed on the left anterior orifice of the surgical template. Implant 4: orthodontic self-drilling mini-implants placed on the left posterior orifice of the surgical template

In addition, the paired *t*-test did not find any statistically significant deviations (*p* = 0.27) between the planned and actual surgical positions of the orthodontic self-drilling mini-implants placed through surgical template at the angular level (Fig. 6).

## Discussion

The results of the present study accept the null hypothesis (H_0_) that there are no differences in the accuracy of the orthodontic mini-implants placement for MSE appliances. Indeed, no statistically significant differences were shown between the coronal entry-point, apical end-point and angular deviations between virtual and actual surgical template of the orthodontic self-drilling mini-implants.

MSE procedures have been highlighted as a promising therapeutic approach for correction of transversal maxillary deficiencies in adult patients [4]. Specifically, Liu et al. reported that 57.5% of the mid palatal suture from a close state after palatal expansion [4]. Furthermore, Hartono et al. showed that posterior locations of the MSE appliance did not lead a more opening of the posterior region [8]. Moreover, bicortical orthodontic self-drilling mini-implants allow more transversal expansion and also higher stability than monocortical orthodontic self-drilling mini-implants [19].

However, MSE procedures require a thorough evaluation of the synosteosis of the palatal suture to assess the expansion prognosis, and additionally the orthodontic self-drilling mini-implants placement site [20]. Moreover, some authors have demonstrated that the palate is a suitable placement site for skeletal anchorage [13, 21, 22] using orthodontic self-drilling mini-implants placed in the paramedian palate to support orthodontic devices [19]. Specifically, palate results a safe placement site for orthodontic self-drilling mini-implants due to the absence of dental roots; however, palate does not present a uniform thickness, which leads the clinician to an accurate planning of bone availability [23, 24] that may influence the primary stability of orthodontic self-drilling mini-implants and thus the success rate and prognosis of MSE procedures [25]. Therefore, computer-aided navigation techniques have been suggested to transfer the digital planning to the clinical setting using surgical templates fixed to anatomical structures [26]. These surgical guides can reduce the discomfort of the patient, avoid damaging anatomical structures during orthodontic mini-implant insertion and therefore, the procedure becomes more conservative, more accurate, safer and faster. They have also been used in numerous other dental procedures such as endodontics, dental surgery etc. [27]. In addition, Bae et al. reported that orthodontic mini-implants placed by computer-aided static navigation technique showed more accuracy than free-hand conventional placement technique; specifically, the computer-aided static navigation technique showed a mean angular deviation of 3.14° (range between 1.02and 10.9 degrees), a coronal entry-point deviation of 0.29 mm (range between 0.03 and 0.73 degrees) mm and an apical end-point deviation of 0.21 mm (range between 0.03 and 0.97 degrees) [28]. However, Liu et al. reported that orthodontic mini-implants placed by computer-aided static navigation technique showed a mean angular deviation of 1.2 ± 0.43° and an apical end-point deviation of 0.42 ± 0.13 mm [11]. This could be due to the stability and inherent support of the surgical template [29, 30] and the threshold value of the radiographic template which may affect the accuracy of the STL model [31]. Additionally, Morea et al. reported that orthodontic mini-implants placed by computer-aided static navigation technique showed a mean angular deviation of 1.76°, a coronal entry-point deviation of 0.86 mm and an apical end-point deviation of 0.87 mm [12]; however, these surgical templates were supported to the teeth and also the bone, and the present surgical template design was only fixed to the bone to prevent deviations derived to the cement procedure to the bands, this could be considered a limitation of the present study; in addition, the surgical templates were even fixed to the completely edentulous upper maxilla with three fixation mini-implants (one anterior and two posterior) to the buccal cortical plate; furthermore, the orthodontic mini-implants where placed monocortical in the interradicular space with direct view and accessibility. In the present study, the results showed that surgical guide and bicortical self-drilling mini-implant placement can achieve the accurate positioning planned. However, Cassetta et al. reported a significant linear correlation between angular deviations and bone density [32]; therefore, higher bone density of bicortical anchor orthodontic self-drilling mini-implantsmay influence higher deviations compared to monocortical anchor orthodontic self-drilling mini-implants.

Recently, Cantarella et al. proposed a making-decision guidelines for orthodontic mini-implant placement using MSE appliances, based on a the virtually 3D design of the orthodontic self-drilling mini-implant placement in the palate; afterwards, the surgical template is also virtually designed, and 3D printed. Finally, the MSE appliance is placed in the oral cavity and orthodontic self-drilling mini-implants are inserted [33]. In addition, Lo Giudice et al. [6] used the method suggested by Cantarella et al. [33] to allow a more accurate discrimination of cortical bone from cancellous bone.

The present study was performed under controlled in vitro conditions and orthodontic self-drilling mini-implants were placed by a certified surgeon with ten years of experience. However, further studies should be performed, preferably in vivo, so that the results can be reinforced.

The results of this study encourage planning the placement of bicortical orthodontic self-drilling mini-implants for the treatment of maxillary skeletal expansion, through a radiodiagnostic study based on a cone beam computed tomography a specific 3D implant-planning software and the manufacture of a surgical template; since it results more accurate than the conventional free-hand technique.

## Conclusion

- Computer-aided static navigation technique enables accurate orthodontic mini-implant placement for the MSE appliances.
- There was no statistically significant differences between the planned and performed orthodontic self-drilling mini-implants at coronal entry-point (*p* = 0.13), apical end-point (*p* = 0.41) and angular deviations (*p* = 0.27).

## Data Availability

The datasets used and/or analysed during the current study are available from the corresponding author on reasonable request.
